# Airway Segmentation and Centerline Extraction from Thoracic CT – Comparison of a New Method to State of the Art Commercialized Methods

**DOI:** 10.1371/journal.pone.0144282

**Published:** 2015-12-11

**Authors:** Pall Jens Reynisson, Marta Scali, Erik Smistad, Erlend Fagertun Hofstad, Håkon Olav Leira, Frank Lindseth, Toril Anita Nagelhus Hernes, Tore Amundsen, Hanne Sorger, Thomas Langø

**Affiliations:** 1 Dept. Circulation and medical imaging, Norwegian University of Science and Technology (NTNU), Trondheim, Norway; 2 Bio-Mechanical Engineering, Faculty of Mechanical Engineering, Delft University of Technology, Delft, Netherlands; 3 Dept. Computer and Information Science, NTNU, Trondheim, Norway; 4 Dept. Medical Technology, SINTEF, Trondheim, Norway; 5 Dept. Thoracic Medicine, St. Olavs Hospital, Trondheim, Norway; Technion - Israel Institute of Technology, ISRAEL

## Abstract

**Introduction:**

Our motivation is increased bronchoscopic diagnostic yield and optimized preparation, for navigated bronchoscopy. In navigated bronchoscopy, virtual 3D airway visualization is often used to guide a bronchoscopic tool to peripheral lesions, synchronized with the real time video bronchoscopy. Visualization during navigated bronchoscopy, the segmentation time and methods, differs. Time consumption and logistics are two essential aspects that need to be optimized when integrating such technologies in the interventional room. We compared three different approaches to obtain airway centerlines and surface.

**Method:**

CT lung dataset of 17 patients were processed in Mimics (Materialize, Leuven, Belgium), which provides a Basic module and a Pulmonology module (beta version) (MPM), OsiriX (Pixmeo, Geneva, Switzerland) and our Tube Segmentation Framework (TSF) method. Both MPM and TSF were evaluated with reference segmentation. Automatic and manual settings allowed us to segment the airways and obtain 3D models as well as the centrelines in all datasets. We compared the different procedures by user interactions such as number of clicks needed to process the data and quantitative measures concerning the quality of the segmentation and centrelines such as total length of the branches, number of branches, number of generations, and volume of the 3D model.

**Results:**

The TSF method was the most automatic, while the Mimics Pulmonology Module (MPM) and the Mimics Basic Module (MBM) resulted in the highest number of branches. MPM is the software which demands the least number of clicks to process the data. We found that the freely available OsiriX was less accurate compared to the other methods regarding segmentation results. However, the TSF method provided results fastest regarding number of clicks. The MPM was able to find the highest number of branches and generations. On the other hand, the TSF is fully automatic and it provides the user with both segmentation of the airways and the centerlines. Reference segmentation comparison averages and standard deviations for MPM and TSF correspond to literature.

**Conclusion:**

The TSF is able to segment the airways and extract the centerlines in one single step. The number of branches found is lower for the TSF method than in Mimics. OsiriX demands the highest number of clicks to process the data, the segmentation is often sparse and extracting the centerline requires the use of another software system. Two of the software systems performed satisfactory with respect to be used in preprocessing CT images for navigated bronchoscopy, i.e. the TSF method and the MPM. According to reference segmentation both TSF and MPM are comparable with other segmentation methods. The level of automaticity and the resulting high number of branches plus the fact that both centerline and the surface of the airways were extracted, are requirements we considered particularly important. The in house method has the advantage of being an integrated part of a navigation platform for bronchoscopy, whilst the other methods can be considered preprocessing tools to a navigation system.

## Introduction

Lung cancer is among the most frequent malignant diseases and overall only 10–15% of patients are expected to survive five years. Early diagnosis and selection of appropriate therapy is essential for survival, as among early stage cancer patients, 38–67% will live for at least five years, compared to 1–8% in the more advanced stages of the disease [1].

The early stage lung cancers are most often small, single tumors in the peripheral parts of the lungs, and they may be hard to reach with biopsy tools in current diagnostic methods. Peripheral parts of the lungs are the locations that the bronchoscope cannot reach, which depends on the diameter of the scope used and the airway lumen. Reaching tumors in the pheriphery also depends on operator skills and the access to navigation.

It is fundamental that the preoperative preparation and planning is optimal in order to enable accurate diagnosis during the first investigation of the patient.

Navigated bronchoscopy is a fairly new diagnostic method that emerged as an attempt to increase the diagnostic success rate for peripheral lung tumours. There are four image-guidance bronchoscopy systems commercially available and nine research and development platforms [2]. In navigated bronchoscopy systems, the position of the bronchoscope and/or biopsy tools are tracked and displayed real-time in a map made from the patient’s preoperative computed tomography (CT) images, providing the operator an endobronchial pathway towards a predefined target, e.g. a lesion. Important technological components of navigated bronchoscopy are the isolation and extraction of anatomical structures of interest from the CT images (segmentation), and the registration of preoperative images to the patient΄s anatomy. Centerline-based registration, one of several options for image-to-patient registration, is a method where the position data from the bronchoscope tip is matched to the center path of the lumen in the bronchi from the preoperative CT images. A recent review state eleven different segmentation methods available for centerline extraction but we consider only the most collective [3].

Region growing is a common method to segment airways from a CT data set [4]. It starts with a predefined seed point and if criteria of intensity threshold are satisfied, adjacent voxels are added to the segmentation. This growing procedure continues until no more valid voxels can be added. Sometimes image noise and other artefacts create holes in the thin wall that separates the airway lumen and lung parenchyma. As these structures have similar voxel intensities in CT images, the region may grow outside the airway lumen. This common problem in region growing algorithms is called leakage.

Segmentation of the airways can be performed by manual, semi-automatic or automatic methods. Manual segmentation is impractical and time-consuming due to the structural complexity of the airways and the dimensions of a typical patient CT data set. The new generation of CT scanners generates a data set of the human airways, which often contain more than 400 slices, each 512x512 pixels [5]. During manual segmentation the user has to perform the entire structure delineation slice by slice. The role of the user in semi-automatic approach is basically reduced to set an initial threshold and place a seed point in one of the slices of the CT data.

The centerline can be extracted from a surface model of the airways by e.g. a thinning algorithm [6]. A different approach is used in the method we developed, the Tube Segmentation Framework (TSF), where first the centerline is detected and then the tubular structure, the airways, “grow” from the extracted centerline [7].

The centerline is used both for alignment of positioning system to CT images [8], but also to indicate the path during navigation. It may also be used for a simplified overview of the airways during navigated bronchoscopy.

Pu et al. [9] presented a review of methods developed primarily for computerized analysis of human airways; they also consider the use of segmentation for path planning in virtual bronchoscopy. Results from manual segmentation are not reliable in a strict sense, since manual delineation typically suffers from relatively large inter- and/or intra reader variability in particular related to small airways [9]. There have been several semi-automatic and automatic methods presented in the literature. Lo et al. [10] presented a review of 15 different methods for bronchial tree segmentation from CT images and today their study is considered a reference for airway segmentation methods. We found studies where the authors mainly compared their own method with a manual segmentation [11–13]. Kiraly et al. [14] compared different segmentation methods but specify few details about the parameters achieved such as number of branches and number of generations. Tschirren et al. [15] also compared other segmentation methods, mentioning number of branches and number of generations achieved but did not compare it to any available commercialized segmentation tool. We have only found two studies that have evaluated the accuracy of segmentation methods in commercially available software. Weissheimer et al. [16] and El et al. [17] presented comparison work between different image processing software for segmentation, but only of the upper airways. None of the studies we found compares results from a commercial system, with segmentation of the bronchial tree including some of the lower peripheral lung segments.

We wanted to examine our own developed method versus commercial software for segmentation of the airways, also for the purpose of seamless preparation, user interaction aspects, and planning and guidance of bronchoscopic procedures. Our work focuses on segmentation of the airways and centerline extraction to be acquired automatically and as quickly as possible before image-to-patient registration and image-guided bronchoscopy, all steps performed in the interventional room. We studied and compared an automatic open access TSF filter [18–19] integrated into our research and development platform for image-guided interventions, CustusX [20], two semi-automatic methods in a commercial system, the Mimics Pulmonology Module (MPM) and the Mimics Basic Module (MBM) (Materialise, Leuven, Belgium) (http://biomedical.materialise.com/mimics), and a semi-automatic method in the freely available DICOM processing application OsiriX [21] (http://www.osirix-viewer.com). We also compared the MPM and the TSF method to the reference segmentation as defined in the study by Lo et al. [10]. As stated on the reference segmentation web page, the images are volumetric chest CT scans acquired at different sites using several different scanners, scanning protocols, and reconstruction parameters. The dataset ranges from clinical dose to ultra-low dose scans, from healthy volunteers to patients with severe lung disease, and from full inspiration to full expiration (http://image.diku.dk/exact/).

The visual representation results from the analysis of our patients’ data were evaluated visually by pulmonologists, radiologists and medical engineers, with emphasis on automaticity and clinically useful segmentations, e.g. complete branches at each generation. We also surveyed the interaction needed to obtain the segmentation. The study should be of interest for those who are considering implementation of navigated bronchoscopy in their clinical practice, in particular with respect to pre-processing of images before intervention.

## Materials and Methods

The anonymized CT data was collected from patients included in a clinical study approved by the Regional Ethical Committee, and all patients signed an informed consent. In the first part of this study we processed 17 CT patient datasets with four different methods in three different software applications; Dynamic region growing provided by MBM, Deep airway segmentation provided by MPM, our own developed TSF method (freely available online) (https://github.com/smistad/Tube-Segmentation-Framework/), implemented in our platform for image-guided interventions, CustusX (an open source platform, SINTEF, Trondheim, Norway) (http://www.custusx.org) and a threshold-based region growing algorithm in OsiriX (Pixmeo, Geneva, Switzerland) (Table 1). Our research group has used OsiriX as a segmentation tool for several years. To obtain airway segmentation in OsiriX the user needs to perform multiple iterations manually to find a proper model mask, which may not always be achievable. Our open source platform for medical image-guided interventions, CustusX, is based on the open access toolboxes VTK (http://www.vtk.org) and ITK (http://www.itk.org).

**Table 1:** Software used in this study.

Software	Description	Operating system	Method / Algorithm
Mimics	Version 15.01 Materialise, Leuven, Belgium Commercial	Windows	Dynamic region growing (Basic Module) (MBM)
Mimics	Version 15.01 Materialise, Leuven, Belgium Commercial	Windows	Deep airway segmentation (Pulmonology Module) (MPM)
OsiriX	Version 5.5, 64 bit ^a^ Pixmeo, Geneva, Switzerland Freely available	Mac OS	Region growing threshold-based algorithm
CustusX	Version 3.5.0 SINTEF, Trondheim, Norway Research platform in development	Ubuntu Linux	Tube Segmentation Framework (TSF) ^b^

^a^ The 32 bit version is free.

^b^ Freely available online

To compare the feasibility of the different solutions we registered the amount of user interaction as number of clicks needed by the operator. The segmentation results were measured as number of branches, total length of branches (without discontinuation), and number of branches segmented for each generation and total volume of the segmented airways.

In the second part of the study we compared our results from the MPM and the TSF methods to the reference segmentation standard by Lo et al. [10]. The reference segmentation is based on 20 different patient datasets with difference in acquisition parameters such as slice thickness, scanner type, image quality and electronic emittance. By following the reference segmentation protocol on their webpage one can evaluate the results to other segmentation methods (http://image.diku.dk/exact/).

The results are interpreted in main three performance measurements: branch detection, tree length detected and false positive rates. Included are also leakage count and volume i.e. voxels outside the correct volume.

1. Branch detection: The count and percentage of branches detected correctly *N*_*seg*_ from segmentation with respect to the total number of branches present in the reference *N*_*ref*_ defined as
  $$\frac{N_{seg}}{N_{ref}}\times 100\%$$
2. Branch length: The total tree length and percentage of tree length in the reference i.e. the fraction of tree length that is detected correctly *L*_*seg*_ by the segmentation relative to the total tree length in the reference *L*_*ref*_ defined as:
  $$\frac{L_{seg}}{L_{ref}}\times 100\%$$
3. False positive rates: The fraction of the segmented voxels that is not marked as “correct” in the reference, defined as:
  $$\frac{N_{w}}{N_{c}+N_{w}}\times 100\%$$
  Where *N*_*C*_ and *N*_*w*_ are the number of voxels in the segmented airway that overlap with the “correct” and “wrong” regions in the reference, respectively. Note that “unknown” regions in the reference are not included in the calculations of the false positives rates. The trachea is excluded from all measures in the reference segmentation, furthermore for the false positive rate the left and right main bronchi are excluded as well.

The Norwegian Regional Committees for Medical and Health Research Ethics (http://helseforskning.etikkom.no) approved the retrospective use of the CT data used in this study. Written informed consent was given by participants for their clinical CT data to be used in this study. The CT data was anonymized and de-identified before data analysis.

### Software used in this study

#### Mimics

Mimics is a commercial medical image processing and analysis software. The user can import various data formats such as DICOM and create 3D models of the patient’s anatomy. Mimics provide both basic functionality and an additional Pulmonology module to process the airways in lung CT scans. For our study we used two modules in Mimics, i.e. a Basic Module and the Pulmonology Module. Both modules used in this study must be purchased separately.

#### Mimics Basic Module

**Fig 1:**
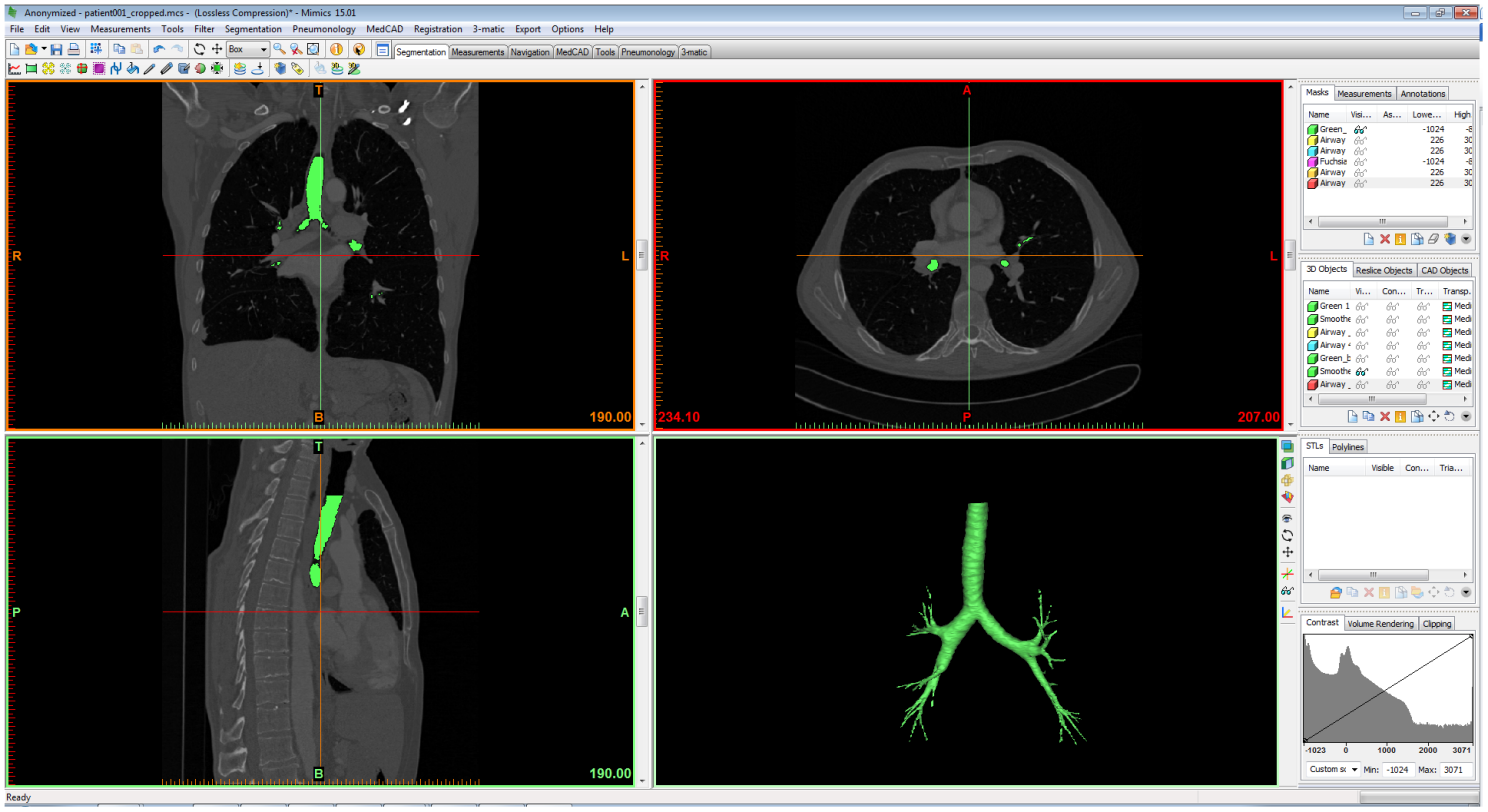
Mimics Graphical User Interface (GUI). In green the result of the segmentation with “Dynamic region growing” in MBM. **A** coronal, **B** axial, **C** sagittal view of the CT data and **D** the 3D model of the airway.

The MBM provides a “Dynamic region growing” segmentation method, which segments an object based on the connectivity of gray scale values in a certain range. The pre-processing starts with manually setting a seed point inside the trachea and selecting a gray value range. For the airway segmentation the minimum value is -990 Hounsfield unit (HU) and the maximum is usually between -50 HU and -120 HU. Trial and error work is needed to find an appropriate range of gray levels that results in a segmentation mask of the airway without leakage. When the mask is set, the option “calculate 3D model from the mask” allows creating a 3D model of the airway that is shown in the right corner of the screen (Fig 1). The model is then smoothed with a smoothing factor (0.9 was used in the example in Fig 1), and a number of iterations factor (1 was used in our cases), which determine respectively how much smoothing and how many smoothing iterations will be performed.

The MBM does not have a tool to extract the centerline, this is provided by a MedCad module, which requires an additional purchase. In this study the centerline of the 3D model made with dynamic region growing has been extracted with the option “centerline labelling” tool of the MPM.

#### Mimics Pulmonology Module

**Fig 2:**
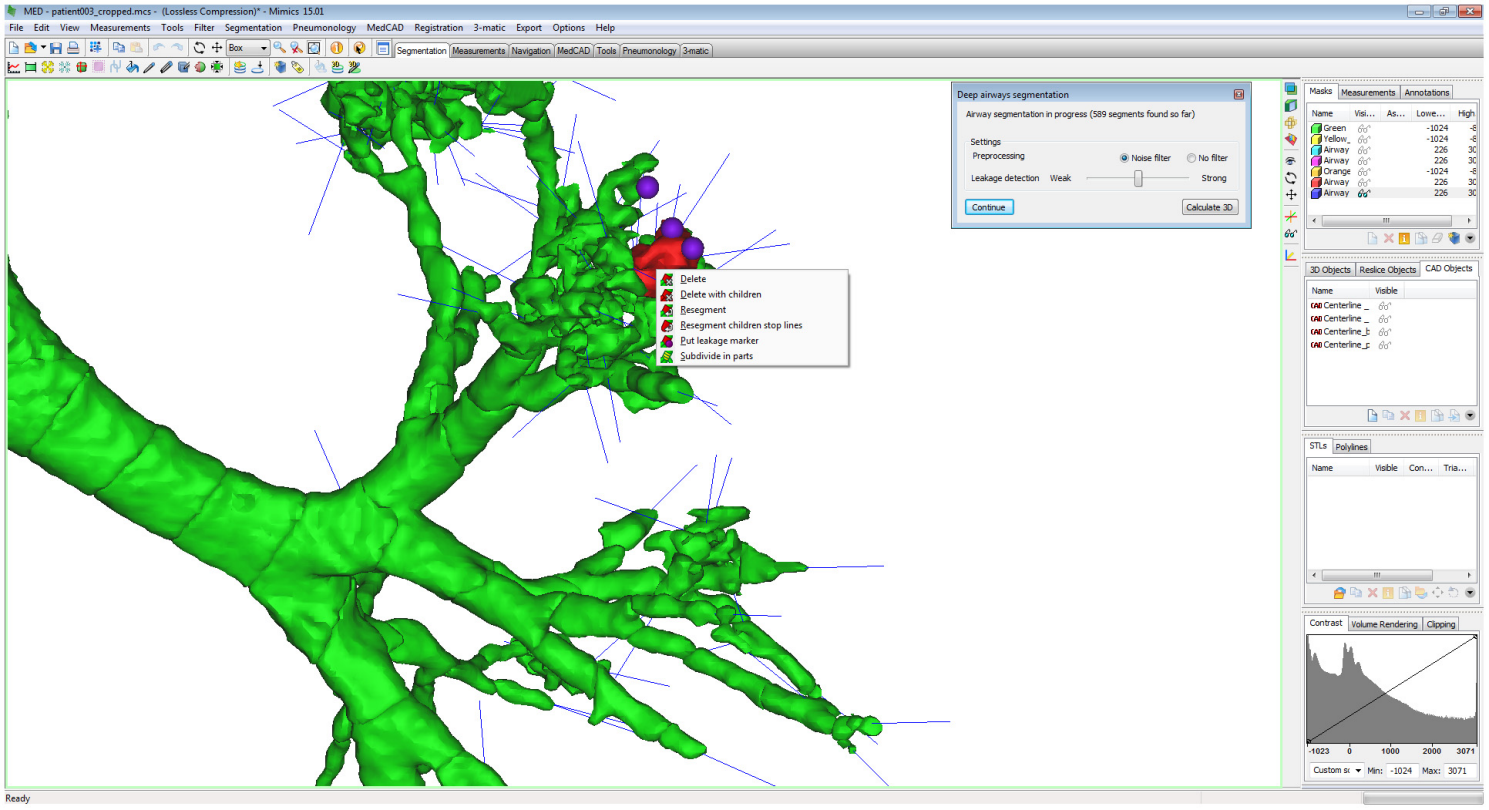
The figure shows a detail of segmentation in MPM. The leakage is well visible at the end of the branches as “clouds” around the branch. The user can select manually the leakage that has to be removed (red segment) and put the leakage marker in its place (purple ball).

The MPM provides the tool “Deep airways segmentation”, where the airways are segmented based on two seed points in the trachea set in the axial view. The first point is the start point of the trachea and by scrolling down a few axial slices from the first slice the second point is set to define the direction of the trachea. After the seed point step, a preview of the airway segmentation appears, and next a 3D model is calculated based on the segmentation mask. During the pre-processing it is possible to select five levels of leakage detection. If the leakage detection level is increased the 3D preview will contain less leakage but the 3D structure will become thinner and have fewer branches, smaller branches will therefore be less likely to be detected. During the following study of the 17 datasets, the level of leakage detection was first put to the third level (default), in order to use as few clicks as possible without leakage. However, if leakage was detected the leakage detection level was increased to fourth or fifth level. In some cases it was not enough to increase the detection level to avoid leakage, consequently requiring some manual work to obtain the most appropriate 3D model possible, by deleting leakages (Fig 2). Leakages may be removed by simply pinpointing (manually clicking) them. The centerline has been extracted by using the tool “centerline extraction”, which also output the name of the branches.

**Fig 3:**
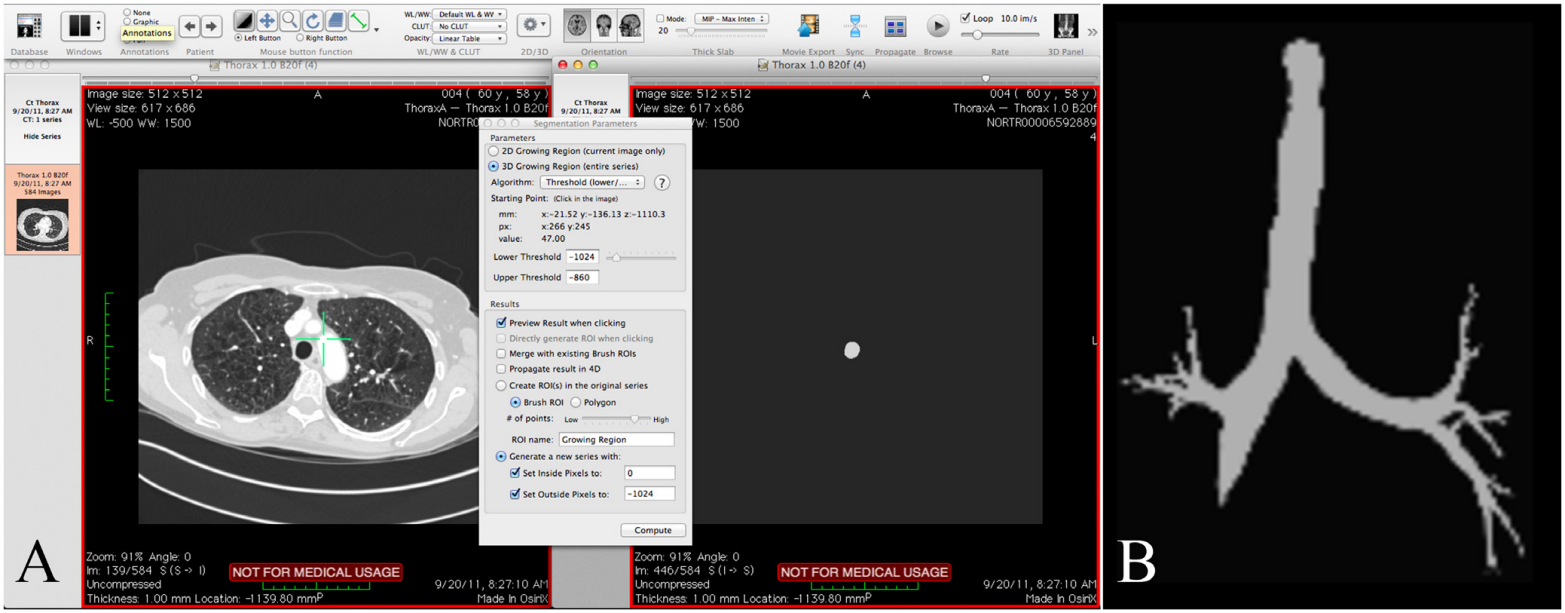
OsiriX GUI. A) axial view of the original CT data, 2D result of the segmentation with threshold upper/lower algorithm, window of the region growing with threshold (upper/lower) algorithm; B) 3D panel.

OsiriX. OsiriX (http://www.osirix-viewer.com) is image processing software for Mac OSX (Apple, Cupertino, USA) dedicated to DICOM image viewing and processing. The OsiriX software offers several algorithms for segmentation; threshold (interval), neighborhood, confidence, threshold (upper/lower), all based on 2D or 3D region growing. Based on initial testing and trial and error we decided to use the Region growing threshold-based (upper/lower) algorithm in our study (Fig 3a). First a range of gray scale values was chosen in order to achieve a segmentation of the airways without leakage. Next, a seed point within the trachea was set and a mask computed. This procedure is time consuming because if the gray scale range chosen is not appropriate, the user has to start over, change the range and compute the mask again. When the 3D model has been obtained, it can be displayed in the OsiriX 3D panel (Fig 3b). A meta-header and raw volume export plug-in was used to export the 3D model for extraction of the centerlines in our own software (CustusX) as OsiriX does not have an option for this.

#### CustusX

**Fig 4:**
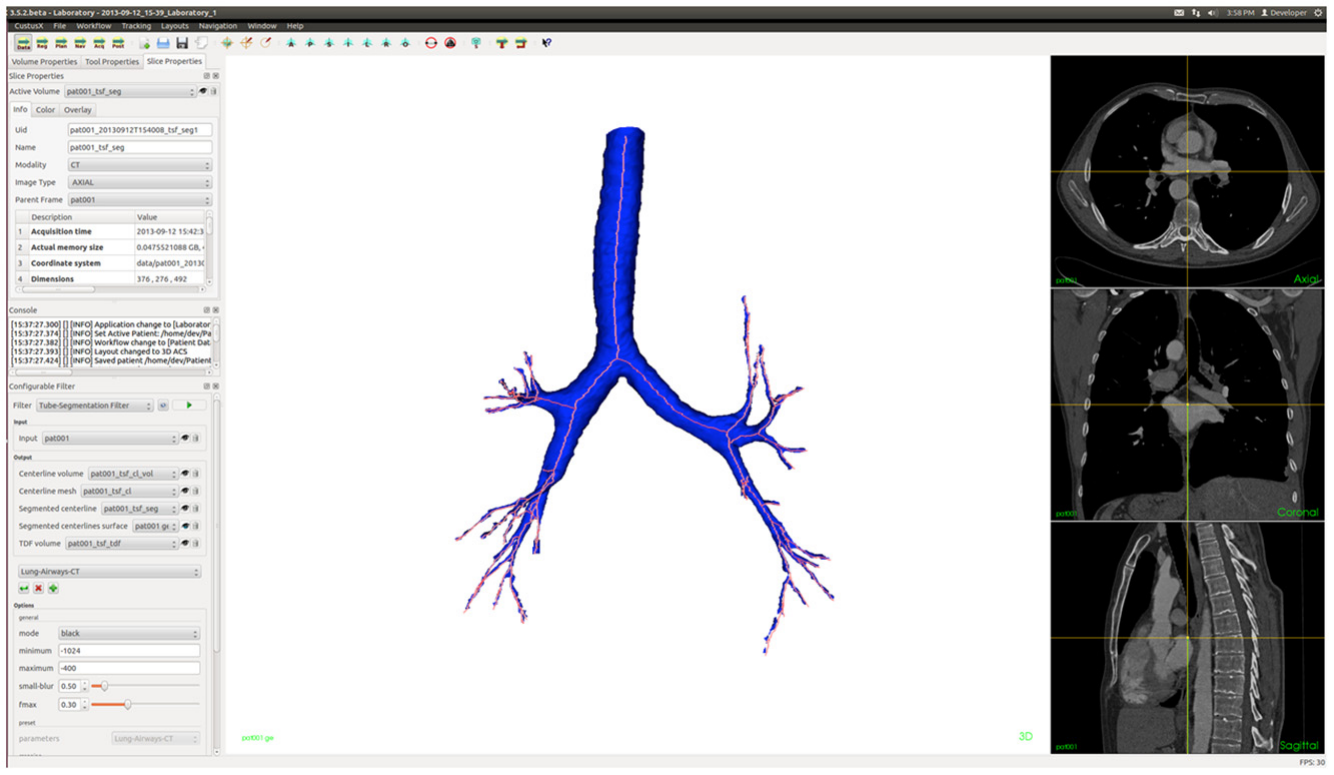
CustusX GUI for TSF. axial, coronal, sagittal view (right side), 3D model of the airway with centerline (center) and configuration filter parameters (left side).

CustusX is a research and development platform for image-guided interventions with focus on intraoperative use [20]. The user can import data in DICOM or meta-header format (.mhd and.raw). The airways are extracted automatically using the open access TSF module (https://github.com/smistad/Tube-Segmentation-Framework/) integrated into the CustusX platform. The TSF, which is the core part being used in this study, uses the graphic processing unit (GPU) to quickly calculate a probability for each voxel whether it is inside a tubular structure or not. First, the method removes data that is not part of the lungs by using a novel automatic cropping algorithm, this is done to reduce memory usage and execution time. Next, a centerline is extracted by automatically selecting a subset of voxels that have a high probability of being inside the airways. These are then linked together to represent the airway structure. Finally, an optional segmentation can be performed by means of a seeded region-growing algorithm using the dilated centerline as seeds and the image gradients to avoid segmentation leakage. Further details of this implementation can be found elsewhere [18–19] (https://github.com/smistad/Tube-Segmentation-Framework/). After the patient CT dataset is imported the user has to choose which parameter preset to use. A parameter preset has to be selected because the TSF can be used to extract blood vessels from different modalities as well as airways from CT. In this case the parameter preset of lung airways was used. The TSF provides the user with both the airways segmentation and centerlines of the airways at the same time (Fig 4).

**Fig 5:**
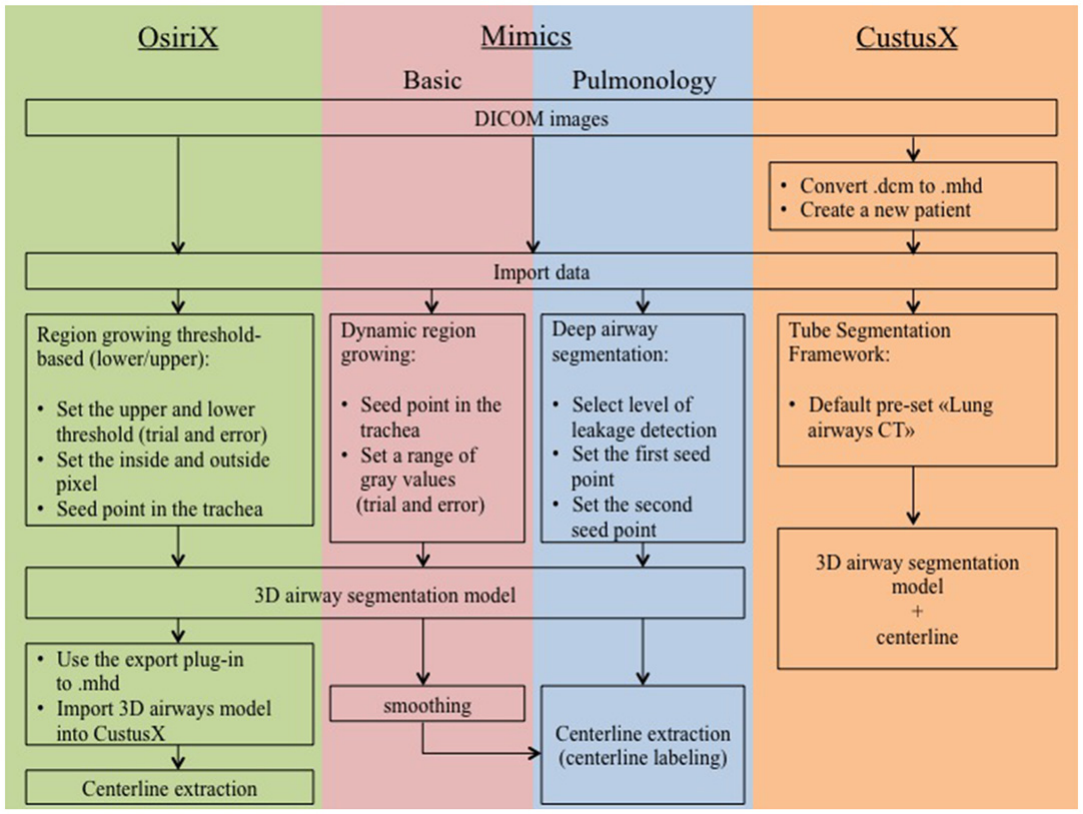
The figure shows a flowchart of analysis of CT data. In OsiriX, MBM, MPM and CustusX.

Parameters used in the study are overall visual inspection and user interaction. Fig 5 summarizes the work performed in each software application used in this study.

### Overall visual inspection

The involved personnel (see author list), initially performed a simple visual inspection of the result to ensure it was anatomically correct and did not contain extensive false positive branches or missing major bronchi in the segmentations. This was performed by visualizing the centerlines and the segmented volumes with the possibility to interactively zoom in and out and rotate the entire structure at the same time as viewing the original CT axial slices.

### User interaction

#### Number of clicks

Operator time is not an exact measure as it depends on the amount of experience the operator has had with the software, therefore we measured the number of clicks in the present study.

**Fig 6:**
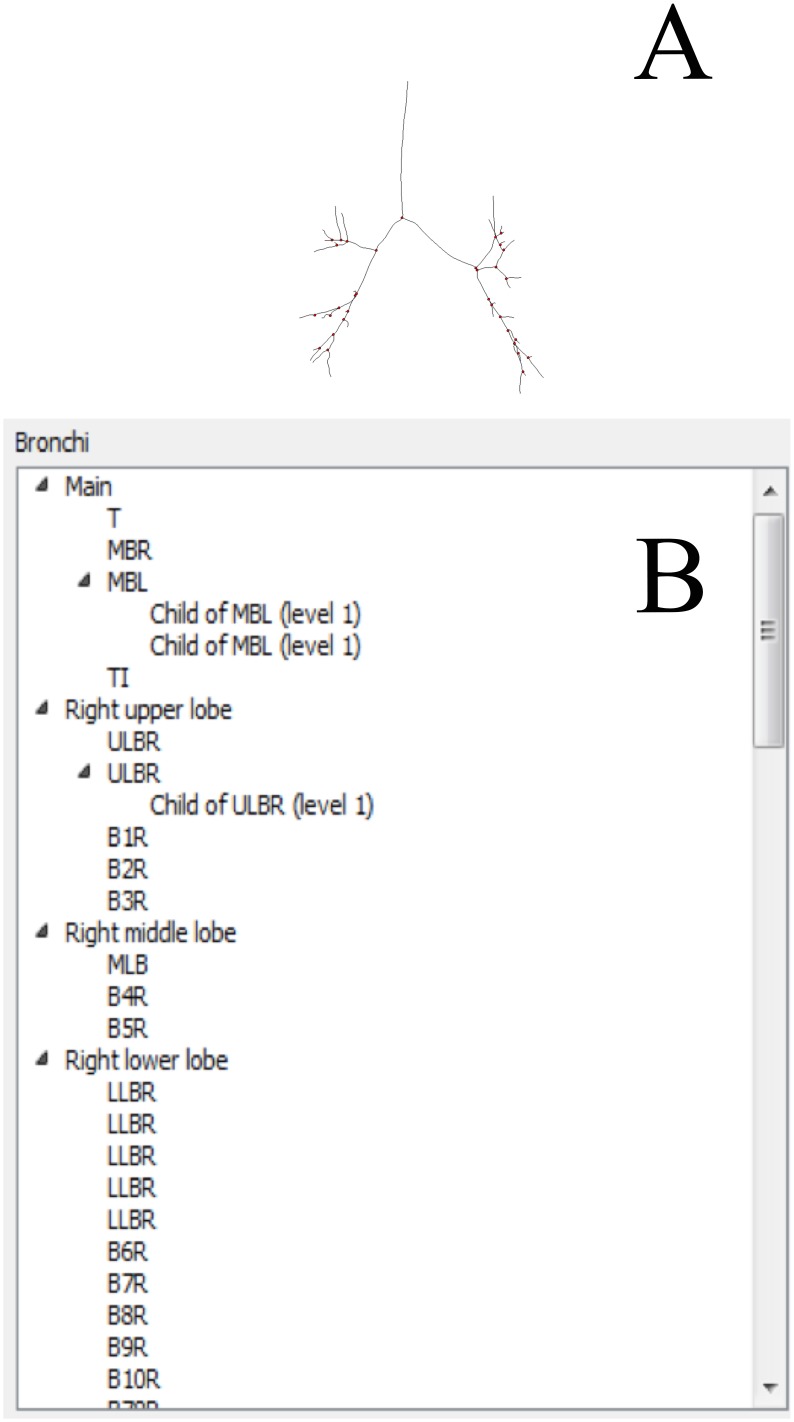
Labeling in Mimics. The figure shows the centerline labeling in MPM. fig B display centerline extracted and fig B list labelling with Mimics.

In Mimics and OsiriX the user can import DICOM CT images directly. For the TSF method integration of CustusX, the CT data had to be converted into meta-header (.mhd and.raw) format, with a simple file format converter, at the time of comparison. In the newest version of the CustusX platform there is a possibility of importing DICOM images directly. Dynamic region growing in MBM demands manual and iterative manual input in order to find the appropriate range of gray scale values for the segmentation. This is a procedure of trial and error that requires a certain number of clicks, which is different for each patient. The MBM does not have the option of centerline extraction. In this study we used the centerline-labeling tool provided by the MPM (Fig 6).

Deep airway segmentation in MPM allows the user to choose the level of leakage detection, which is medium as default (third level). Additional clicks are needed when the user decides to change the level. In our study the fourth level was chosen for five of the patients and level five for three of the patients. This was done to reduce or eliminate the leakage in the final 3D model. Leakages in five patients were removed manually; in this case the number of clicks increases depending on the quantity of leakage to be deleted.

OsiriX does not have the option for centerline extraction. The airways segmented with OsiriX were imported into CustusX where the centerlines were extracted using a thinning algorithm.

#### Segmentation

Included number of branches, number of generations and total length displayed. Text files with coordinates of the centerlines from Mimics were processed in MATLAB (Mathworks Inc., USA) to calculate the number of branches, number of generations and total length of the centerline of the segmented airway branches. MATLAB was also used to process the Visualization Toolkit (VTK) files from CustusX to obtain the parameters of the 3D model and centerline obtained from the TSF in CustusX. The actual number of branches for each of the five first generations in a few control datasets was found by manually identifying airway bifurcations in the CT volumes.

#### Volume

The first step in the TSF method used in CustusX is to crop the dataset in order to save processing time. Each dataset contained between 241 and 843 slices with a percentage of reduction in number of slices from 5.65% to 29.03%. The slice increment was 0.5 mm, except in one patient, where it was 1.25 mm. This information was used to set the same starting point in the trachea in the other two software applications.

Dynamic region growing in the MBM creates a mask, which is then cropped with the tool “crop mask”. The crop is automatically performed with the TSF method. The same crop is performed on the data before processing in Mimics in order to have comparable results for the different methods. The volume was calculated in the software directly after the 3D model had been created. In the MPM we set the same starting point as used in the Basic module and the volume is provided directly from the user interface of the software. In OsiriX we imported the dataset starting with the first (axial) slice after the cropping position. After segmentation, we were not able to measure the volume directly from the software or with other methods. The volume of the airways in TSF method was calculated from the number of voxels multiplied by the volume of a single voxel. This procedure was automated with a simple MATLAB script.

A total of 51 measurements of each parameter were made. To evaluate the correlation between the software, the Pearson correlation coefficient was used.

## Results

### User interaction

#### Number of clicks

The number of clicks needed to import the data for both Mimics modules were six, in OsiriX four, and in TSF it was 14. If we do not count the extra clicks needed to convert the file format for TSF, the number of clicks for importing the data was six. This is the case for the newest version of CustusX used for TSF, but an older version without DICOM import was used in this study. MPM demanded different number of clicks for the segmentation procedure depending on the level of leakage detection chosen. Seven clicks were necessary to provide segmentation and centerlines for TSF. The number of clicks for both Mimics modules does not include the process of cropping which can increase the number of clicks by nine clicks. All the results are summarized in Table 2.

**Table 2:** Number of clicks, mean ± standard deviation needed to import data and, perform a segmentation and centerlines extraction.

Software	Import	Segmentation and centerline extraction	Total
MBM	6	22 ± 4	28 ± 4
MPM*	6	11^a^	17
MPM*	6	17^b^	23
MPM*	6	24^c^	30
MPM*	6	33 ± 11^d^	39 ± 11
OsiriX	4	41 ± 4	45 ± 4
TSF (CustusX)	14	7	21

* In MPM we distinguished between four cases:

^a^Segmentation with the third level of leakage detection;

^b^Segmentation with the fourth level of leakage detection;

^c^Segmentation with the fifth level of leakage detection;

^d^Segmentation with leakage, we need to clean it manually.

#### Segmentation

**Fig 7:**
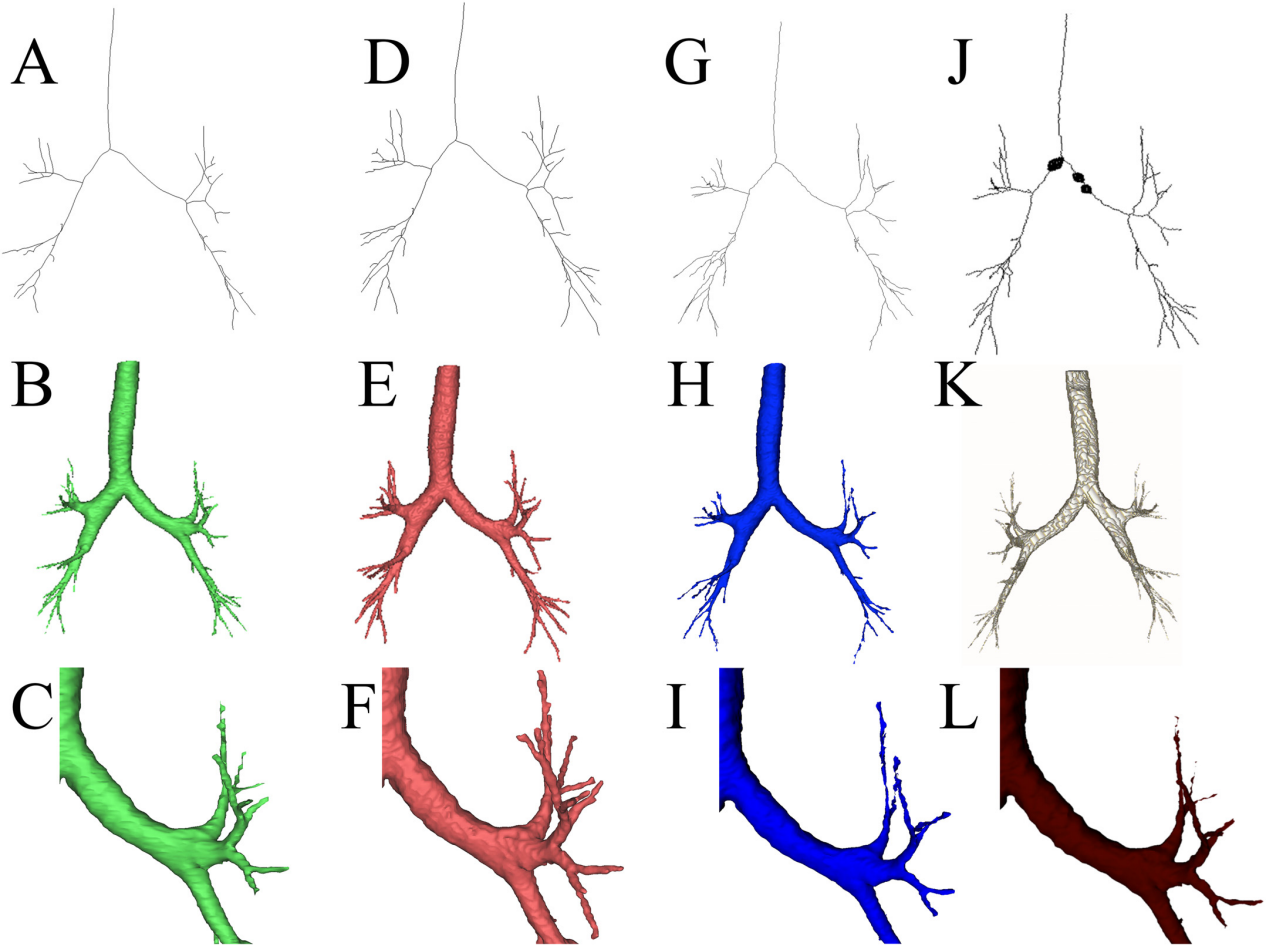
The figure shows segmentation, centerline, model and a detail of the airways of patient #1 in MBM (A,B,C), MPM (D,E,F), TSF(G,H,I) (CustusX) and OsiriX(J,K,L) respectively (left to right). The centerline for Osirix was produced in CustusX by using the standard filter for centerline extraction (thinning algorithm). In the centerline of OsiriX there are three black circles caused by holes in the segmentation.

Examples of segmentation and centerline of patient #1 are shown in Fig 7.

Following results for number of branches, total length and number of generations gave following. The mean and standard deviation of the total length of airways branches in millimeters (mm) in MBM, MPM and TSF were, respectively, 954 ± 406 mm, 1261 ± 565 mm and 806 ± 208 mm. The centerlines were not possible to extract in three patients (10, 12 and 14) in MBM due to the presence of holes in the 3D model. The correlation factor for the total length was 0.944 between the two modules in Mimics, 0.590 between MBM and TSF and 0.633 between MPM and TSF.

The average highest generation found by using MBM was 8.28 ± 1.43 and the average total number of branches was 59.42 ± 28.20, in MPM the highest generation was 9.53 ± 2.00 and 81.00 ± 40.50 branches were segmented, and in TSF the highest generation was 7.06 ± 1.60 and 41.52 ± 15.42 branches were segmented. In the four control datasets, where the actual number of branches was manually counted for the first five generations, all methods found all branches up to the fourth generation (± one branch for the fourth generation) for three of the datasets. In the data from patient 17, MBM missed branches from the third generation and all methods missed branches from the fourth generation. All methods missed branches at the fifth generation for all control datasets, MBM found 53% of the fifth generation branches, MPM 69% and TSF 51%.

The segmentations from OsiriX were sparse and that made it nearly impossible to get true centerlines of the airways. For only three of the patients it was possible to obtain the centerlines from the segmentation in OsiriX.

**Fig 8:**
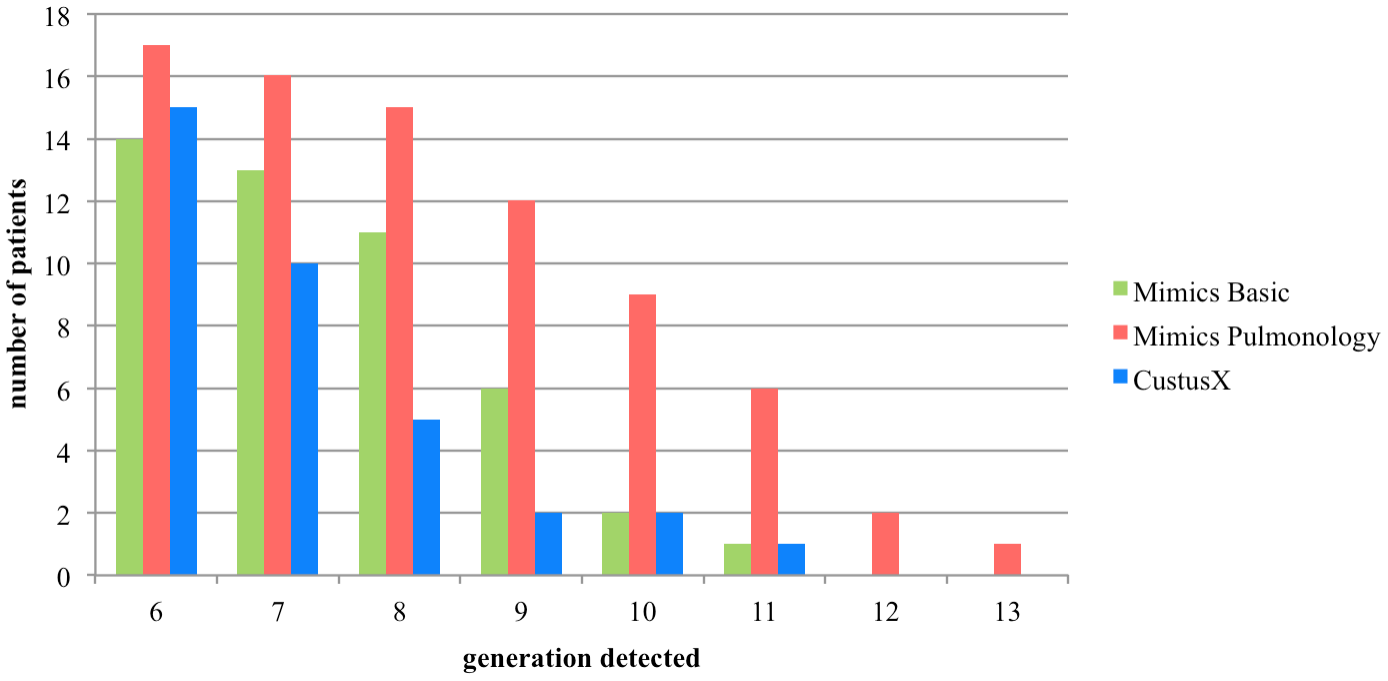
Number of patients in each generation. The plot shows the number of patients that each software is able to detect, up to the sixth generation.

Method comparison based on number of branches in each generation for all patient cases is presented in Table 3. Fig 8 shows for how many patients each method is able to detect branches of each generation, starting from the sixth generation.

**Table 3:** Method comparison, number of branches in each generation for all patients.

Patient number	Software						Generation							
		1	2	3	4	5	6	7	8	9	10	11	12	13
**1**	MBM	1	2	5	12	16	8	4	6					
**1**	MPM	1	2	5	12	20	12	12	10	6				
**1**	TSF	1	2	5	13	9	4	2	2					
**1**	Manual counting	1	2	5	13	27								
**2**	MBM	1	2	4	8	14	22	12	4	2				
**2**	MPM	1	2	4	8	14	16	15	7	2				
**2**	TSF	1	2	4	9	13	10	4	4					
**3**	MBM	1	2	4	9	19	29	20	16	10	6			
**3**	MPM	1	2	4	8	17	29	38	19	14	8	2		
**3**	TSF	1	2	4	9	20	17	10	5	4	2			
**4**	MBM	1	2	4	8	10	8							
**4**	MPM	1	2	4	6	10	16	4	2					
**4**	TSF	1	2	4	8	5	4							
**5**	MBM	1	2	4	6	4	4	2						
**5**	MPM	1	2	4	6	4	6							
**5**	TSF	1	2	4	8	4								
**6**	MBM	1	2	4	9	12	23	12	9	2	2			
**6**	MPM	1	2	4	9	16	24	24	16	8	4	2		
**6**	TSF	1	2	4	8	14	21	15						
**6**	Manual counting	1	2	4	8	17								
**7**	MBM	1	2	4	8	14	22	12	8	4	4	4		
**7**	MPM	1	2	4	8	14	24	30	18	12	4	6	2	2
**7**	TSF	1	2	4	9	13	12	7	6					
**8**	MBM	1	2	4	10	20	20	15	8	4				
**8**	MPM	1	2	4	9	18	32	27	17	7	6	4		
**8**	TSF	1	2	4	10	13	9	3						
**9**	MBM	1	2	4	6	8	4	4						
**9**	MPM	1	2	4	6	8	10	10	8	6	2			
**9**	TSF	1	2	4	6	12	10							
**10**	MBM													
**10**	MPM	1	2	4	6	12	12	10	6	4	6	6	2	
**10**	TSF	1	2	4	9	12	9	2						
**11**	MBM	1	2	4	8	14	22	14	6					
**11**	MPM	1	2	4	8	16	24	24	16	6	4			
**11**	TSF	1	2	4	6	6	6							
**12**	MBM													
**12**	MPM	1	2	4	8	17	27	28	14	11	7	2		
**12**	TSF	1	2	4	10	19	11	9	4	2	2	2		
**13**	MBM	1	2	4	9	10	10	4						
**13**	MPM	1	2	4	8	12	12	4	2					
**13**	TSF	1	2	5	10	10								
**13**	Manual counting	1	2	4	9	18								
**14**	MBM													
**14**	MPM	1	2	2	4	9	14	8	4	6				
**14**	TSF	1	2	4	10	16	7							
**15**	MBM	1	2	4	8	16	20	21	12	2				
**15**	MPM	1	2	4	8	17	26	32	29	15	2			
**15**	TSF	1	2	4	9	16	5							
**16**	MBM	1	2	4	8	17	8	2						
**16**	MPM	1	2	4	8	12	6							
**16**	TSF	1	2	4	8	13	4	2						
**17**	MBM	1	2	2	5	6	6	2	2					
**17**	MPM	1	2	4	6	9	6	4	2					
**17**	TSF	1	2	4	8	9	9	6						
**17**	Manual counting	1	2	4	10	21								

**Fig 9:**
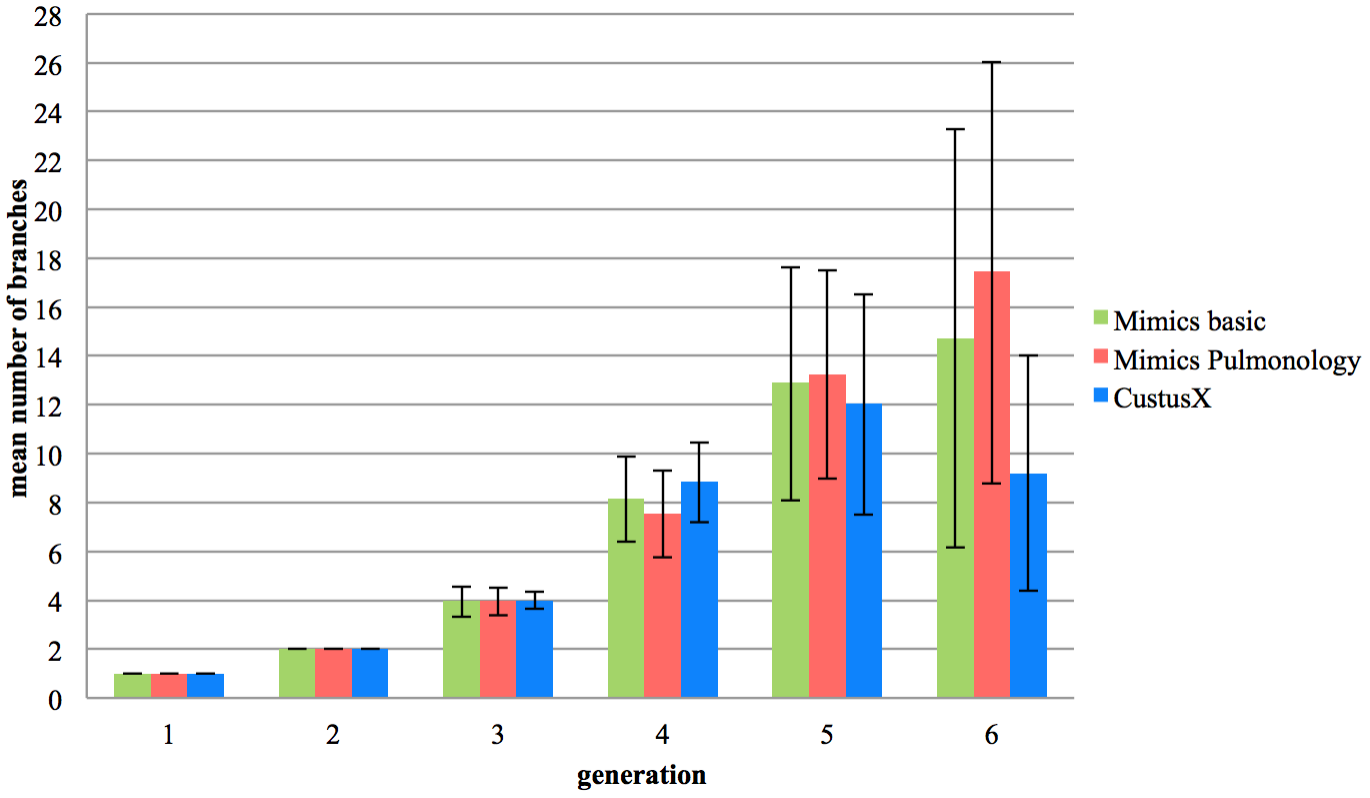
Average branches detected in each generation. The plot shows the mean and standard deviation of number of branches per generation for each method.

The mean and the standard deviation of the number of branches segmented per generation (until sixth) detected by each software are reported Fig 9.

#### Volume

The mean and standard deviation of the volume was found to be 37736 ± 15948 mm^3^ in MBM, 46759 ± 20511 mm^3^ in MPM and 34302 ± 15346 mm^3^ in TSF. The segmentation of one patient (#12) in MBM was sparse making it impossible to achieve a measure of the volume. The reason why Dynamic region growing in MBM failed with this patient data might be due to the presence of a tumor. The correlation factor was 0.98 between the two modules of Mimics, 0.99 between MBM and TSF, and 0.98 between MPM and TSF.

### Reference segmentation

A reference segmentation comparison from MPM and our in-house method are shown in Table 4 with computed overall reference segmentation averages and standard deviations for branch count, branch detection percentage, percentage for path length detection, leakage count, leakage volume and percentage of false positive rate.

**Table 4:** Reference segmentation results, comparison for MPM and TSF method, one can compare to results in EXACT09 webpage (http://image.diku.dk/exact/).

	Branch count	Branch detected (%)	Tree length (cm)	Tree length detected (%)	Leakage count	Leakage volume (mm3)	False positve rate (%)
Mean (MPM)	104.7	42.9	78.7	37.5	4.4	120.4	0.9
SD (MPM)	55.2	9.6	41.7	7.1	5.9	249.2	1.6
Mean (TSF)	72.4	31.3	54.3	27.4	34.5	344.8	3.6
Std. dev. (TSF)	37.8	10.4	33.9	9.6	23.4	397.8	3.4
Average (Overall for reference segmentation)	124.0	51.8	95.0	46.0	9.8	700.6	2.8
Std. dev. (Overall for reference segmentation)	62.9	13.5	52.1	12.9	10.1	856.1	3.4

The mean and standard deviation for the branch detection were 42.9±9.6% and 31.3±10.4% for the MPM and the TSF methods, respectively, both lower than the reference segmentation average but within the standard deviation range of 51.8±13.5%. Mean and standard deviation for the tree length detected was 37.5±7.1% and 27.4±9.6% for the MPM and the TSF methods, respectively, both lower than the reference segmentation average but within the standard deviation range of 46.0±12.9%. Mean and standard deviation for the leakage count was 4.4±5.9% and 34.5±23.4% for the MPM and the TSF methods, respectively, compared to reference segmentation average of 9.8±10.1. The mean and standard deviation for the leakage volume was 120.4±249.2 mm^3^ and 344.8±397.8 mm^3^ for the MPM and the TSF methods, respectively, are both lower than the reference segmentation average of 700.6±856.1 mm^3^. The mean and standard deviation for the false positive rate was 0.9±1,6% and 3.6±3.4% for the MPM and the TSF methods, respectively, which is at a similar level as the reference segmentation average of 2.81±3.3%.

## Discussion

We have compared three different software applications for lung airways segmentation and centerline extraction and evaluated their performance. Overall, the results show that all the applications were feasible for processing lung CT images, but the applications differed regarding completeness of segmentation, automaticity and user interactions.

The ideal approach, according to the literature, for the assessment of the airway segmentation is using a manual segmentation as a reference [9]. This was not possible in our case due to the time and the complexity of such a task. Another accepted approach is the reference segmentation, by Lo et al. [10], which we used successfully for comparison in this study. Both MPM and TSF are compatible with the overall for reference segmentation averages and standard deviations of the reference segmentation except for the leakage number and volume that TSF got.

In addition, the segmentation methods were investigated using our own CT data from 17 patients. The number of branches for each generation was automatically counted and the volume was found. As a control we also performed a manual count of the number of branches for each of the first five generations in four selected control data sets. Counting beyond the fifth generation would be technically challenging and time consuming, and it would not add much extra information as none of the segmentation methods were able to identify all branches of the fifth generation, thus, none would be able to identify all branches of higher generations either. For navigated bronchoscopy it is also of higher importance to reveal if a method is able to identify, segment and find the centerline of a branch, than that the exact volume and branch radius is found. The total length of airways branches in millimeters (mm) depends on the number of branch detected.

### User interaction

#### Number of clicks

The main goal of our work is the use of 3D airways model visualizations for guidance in navigated bronchoscopy. In this work we are seeking a seamless procedure with as little necessary user interaction as possible to be able to use guiding techniques in as many patients as possible. The preparations for each case should therefore be quick and as automatic as possible. Hence, limited user interaction, measured in clicks, are important in a clinical setting.

The segmentation in OsiriX and Mimics, particularly the MBM, require a manual and iterative procedure in order to set the right range of gray scale values, i.e. the user has to change the range until there is no leakage into the lung parenchyma. With Dynamic region growing in Mimics the results are displayed in the CT images immediately while one changes the upper and lower gray scale value, but with OsiriX the user needs to change settings and manually iterate to obtain the most appropriate results from the user’s personal perspective. Changing one setting, the result in one 2D slice can be viewed and the slice can be changed, but the 3D result is only visible after running the segmentation with a chosen setting. During the processing, no feedback is provided, and the user has to “wait and see”. This might have to be done several times, until the 3D model is segmented without leakage.

After the choice of the two seed points in MPM the segmentation mask is created automatically. If the results show leakages, some manual work is necessary to “clean” the model. The user decides when to stop by visual analysis of the model.

The segmentation process of the TSF in CustusX is fully automatic and the user interaction is reduced to only importing data and choosing the parameter preset for the structure of interest, e.g. CT airways. The parameter set for each type of image modality (CT/MR) and organ combination can be optimized and set for all future cases [20]. These parameters have been optimized manually.

The MPM is the software that demands fewest clicks to achieve a segmentation of the airways and their centerlines. However, this is not the case when we need to increase the level of leakage detection or when the result presents leakage that has to be removed manually. TSF on the other hand requires more clicks than the other methods to import the data since the data format has to be changed. If the importing of data is excluded, TSF has the fewest clicks to segment the airways, only seven clicks are needed for segmentation and centerline extraction compared to both modules of Mimics. When performing segmentation of the bronchial tree intraoperatively it is of course an advantage that the user has to perform as few clicks as possible and that the method is as automatic as possible. An intraoperative TSF integrated solution such as CustusX, is less time consuming than a preoperative system, such as Mimics or OsiriX. Nevertheless, robustness and accuracy are important as well.

#### Segmentation and Volume

The MBM failed segmentation in one patient due to a tumor in the trachea. It also failed centerline extraction in three patients due to the presence of holes in the segmentation. TSF and the MPM were both successful in segmenting the airways in all 17 patients CT scans.

The number of branches per generation and highest generation detected, and hence also total length of the airways was largest in the Mimics modules. The highest correlation factor between the measures of the total lengths was between the MBM and the MPM, indicating the similarity of the algorithms in the two modules.

The total number of branches detected in all patients was highest for the MPM, followed by the MBM and TSF. Only in one patient (#17) TSF detected more branches than the Mimics modules. The differences were less evident in the histogram of the number of generations. Both modules in Mimics have the highest generation detected. Nevertheless, TSF is able to achieve the same maximum level of generation as MBM in four patients, and the average highest generation detected between the two methods differs only by one generation. All methods are able to detect the trachea (first generation) and the main carina (second generation), but there are difference in branch number from third generation (Table 3). In seven patients TSF was not able to segment beyond the sixth generation, by comparison to the MPM, where the result was up to eighth generation in more than 50% of the patients. TSF was able to detect more branches of the fourth generation in ten patients compared to both Mimics modules. Compared to both Mimics modules, TSF detected the least number of branches but it has the smallest standard deviation, which means it is more consistent than Mimics in detecting branches between datasets. In four of the datasets branches up to the fifth generation were counted as a control. One dataset (patient #17) appeared to be challenging as none of the segmentation methods found all branches beyond the third generation. In the other three datasets all branches up to the fourth generation were found by all methods. In a few instances the number of branches for the fourth generation was one less or even one more compared to what was found by manual counting. This small inaccuracy is likely caused by an ambiguity whether a bifurcation point connects a branch with three branches of the next generation or two branches where one is very short followed by a new division. This assumption is based on the fact that we inspected the segmentation result for false positive detected branches. None of the methods found all branches at the fifth generation.

The volume results are linked with the results of number of branches and total length of the airways. Branches of the first generations are larger than in the latest generations, therefore they have a higher influence on the total volume. Defined volume of any patient lung data is not known but can give an idea of the difference between them. The MPM and the TSF methods also had lower leakage on average than the reference segmentation.

All software were able to detect branches up to the sixth generation in all the patients, then, since the volume measurement strongly depend on the first generation of the airways, the correlation factors for the volume results are very high. The correlation factor between the software applications is relative due to the fact that the real volume for each airway is unknown. But the difference between the volume measurement from each software changes in appropriate proportion between patient datasets.

The necessary number of branches and to what generation is needed for clinical application depends on the specific clinical case. The smallest diameter of existing bronchoscope technology is 2.4 mm, which is enough to maneuver to the fifth generation, considering that the average diameter in human lungs of apical bronchus and basal bronchus are 2.8 ± 0.6 mm and 2.7 ± 0.6 mm respectively [22]. Steerable catheter added into the navigation can be advanced into sixth-seventh generation, depending on the catheter diameter, which normally is 2 mm. When thinner bronchoscopes enters the market, the accuracy concerning the number of branches in each generation will become more valuable and specially when sampling peripheral lung lesions. Still, it is more important that the method is able to find as many of the bronchial branches as possible up to the sixth generation, compared to finding some of the branches in higher generations.

Table 5 shows advantages and disadvantages of the three different software systems used in this study.

**Table 5:** Advantages and disadvantages between Mimics (Basic and Pulmonology module), CustusX and OsiriX.

Software	Advantages	Disadvantages
Mimics	Quick segmentation, semi-automatic process, large segmentation parameters range	Commercial software (not free, relatively expensive), no navigation functionality
Mimics	Pulmonology module contains all tools needed for segmentation and centerline extraction, better results than Basic module in airways segmentation.	Basic module needs another tool for centerline extraction, manual steps needed to find the scale of gray level and can fail in data extraction.
Mimics	The MPM performed better than CustusX on average, at the same time there is no statistically significant difference on the results for the two methods.	Pulmonology module has additional cost
CustusX	Quick segmentation with automatic process, segmentation and centerline extraction at the same time. Available to collaboration partners via agreement	Need to convert DICOM to internal platform data first (plans to integrate DICOM import)
CustusX	Method more consistent than Mimics (smallest STD values)	Detects less generations and branches in general than Mimics
CustusX	Possible to perform all steps in operational room and navigation platform in same system	
CustusX	Available to collaboration partners via agreement	
OsiriX	Freely available	Manual steps to find the scale of gray levels (trial and error).
OsiriX		Need another software for the centerline extraction
OsiriX		Segmentation time consuming compared to the other software solutions

### Segmentation parameters difference in reference data vs. our patient data

There is a difference in terms between the reference segmentation and our patient data measurement. If there is a correct or true volume not connected to the branch tree we count it within our results but the reference segmentation demands a complete volume and all disconnected volumes from the bigger volume are not counted as results.

### Reference segmentation comparison

The leakage count average and standard deviation for TSF method is higher than the reference segmentation but the leakage volume is low compared to the reference segmentation. The TSF method does thus create an extensive number for small leakages. That demonstrates the usage of the reference segmentation because the TSF method is automatic with no manual segmentation or post-segmentation smoothing algorithm to remove noisy additional voxels. MPM could have post-segmentation smoothing algorithm to ignore small leakages and disconnection of one or a few voxels. Finally the false positive rate averages and standard deviations for MPM and TSF methods are similar to the reference segmentation. The mean and standard deviation for the branch detection were 42.9±9.6% and 31.3±10.4% for the MPM and the TSF methods, respectively. The MPM performsed better than CustusX on average. At the same time there is no statistically significant difference on the results for the two methods.

## Conclusion

The segmentation results from both MPM and TSF are sufficient for navigated bronchoscopy. Segmentation and centerline extraction of the airways are obtained with the least number of clicks in MPM if no leakage occurs. TSF demands more clicks than the other solutions to import the data since the data format has to be changed. Once the data is imported into CustusX, TSF provides a fast and automatic segmentation and centerline extraction, without using any extra manual pre-processing work (setting seed point and/or range of gray values) or time consuming manual post-processing work (removing leakages). OsiriX requires the highest number of clicks to process the data, the segmentation is often sparse and extracting the centerline requires the use of additional software. Mimics can detect a higher number of generations and branches than TSF, while TSF is more consistent in detecting almost the same number of branches in each generation over several data sets. Future work for TSF can be adding post-segmentation smoothing algorithm to remove noisy additional voxels and increase the possibility to detect more airway generations. We believe that navigated bronchoscopy will become more regularly used in lung medicine in the near future. To accomplish integration of such guidance technology it is important that preprocessing of data is precise, fast, and automatic and is integrated in a seamless manner into the workflow. This is particularly true for preoperative work in a busy clinical setting.
